# Supplementary material for: Treatment with the Olive Secoiridoid Oleacein Protects against the Intestinal Alterations Associated with EAE
Source: Int J Mol Sci. 2023 Mar 4;24(5):4977. doi: 10.3390/ijms24054977 (PMC10003427; doi:10.3390/ijms24054977)
Supplement: Supplementary file 1 [file ijms-24-04977-s001.zip › ijms-2149972-supplementary.pdf]

Figure S1

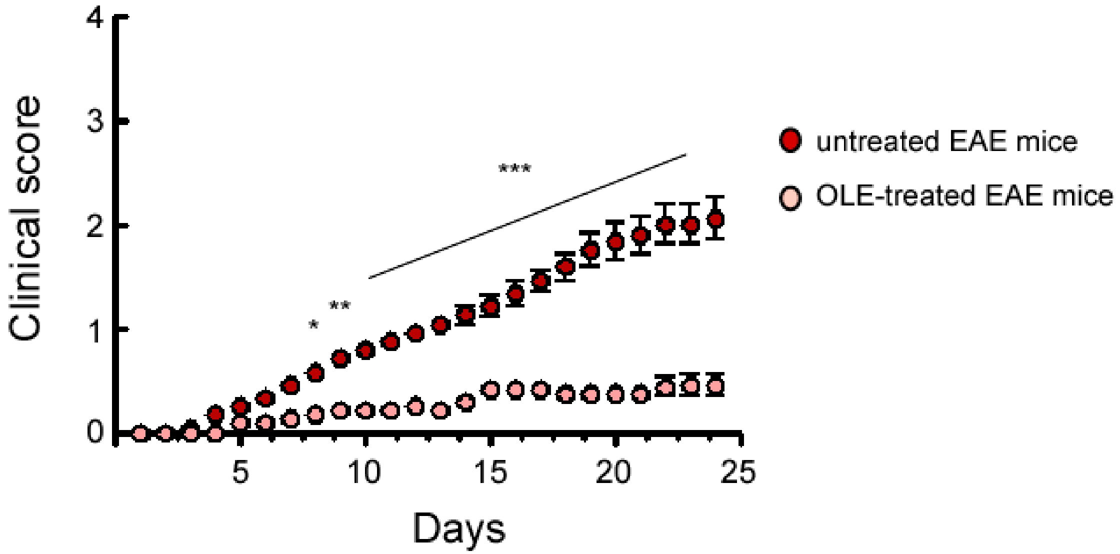

Figure S1. Effect of OLE treatment on clinical parameters in EAE mice.
Effect on the evolution of clinical signs in untreated EAE mice and OLE-treated EAE mice (n= 10, in all groups). *$p<0.05$, **$p<0.01$ and ***$p<0.001$ *vs* OLE+EAE
